# Novel Missense *CACNA1G* Mutations Associated with Infantile-Onset Developmental and Epileptic Encephalopathy

**DOI:** 10.3390/ijms21176333

**Published:** 2020-08-31

**Authors:** Géza Berecki, Katherine L. Helbig, Tyson L. Ware, Bronwyn Grinton, Cara M. Skraban, Eric D. Marsh, Samuel F. Berkovic, Steven Petrou

**Affiliations:** 1 Ion Channels and Disease Group, The Florey Institute of Neuroscience and Mental Health, The University of Melbourne, Parkville, VIC 3052, Australia; 2 Division of Neurology and The Epilepsy NeuroGenetics Initiative, Children’s Hospital of Philadelphia, Philadelphia, PA 19104, USA; helbigk@email.chop.edu (K.L.H.); marshe@email.chop.edu (E.D.M.); 3 Department of Paediatrics, Royal Hobart Hospital, Hobart, TAS 7000, Australia; tyson.ware@ths.tas.gov.au; 4 Epilepsy Research Centre, Department of Medicine, University of Melbourne, Austin Health, Heidelberg, VIC 3084, Australia; grinton@unimelb.edu.au (B.G.); s.berkovic@unimelb.edu.au (S.F.B.); 5 Department of Pediatrics, Perelman School of Medicine at the University of Pennsylvania, Philadelphia, PA 19104, USA; skrabanc@email.chop.edu; 6 Department of Neurology and Pediatrics, Perelman School of Medicine at the University of Pennsylvania, Philadelphia, PA 19104, USA; 7 Department of the Florey Institute, University of Melbourne, Parkville, VIC 3050, Australia

**Keywords:** *CACNA1G* mutation, voltage-dependent T-type calcium channel, developmental and epileptic encephalopathy, deep cerebellar nuclei, gain of function, loss of function

## Abstract

The *CACNA1G* gene encodes the low-voltage-activated Ca_v_3.1 channel, which is expressed in various areas of the CNS, including the cerebellum. We studied two missense *CACNA1G* variants, p.L208P and p.L909F, and evaluated the relationships between the severity of Ca_v_3.1 dysfunction and the clinical phenotype. The presentation was of a developmental and epileptic encephalopathy without evident cerebellar atrophy. Both patients exhibited axial hypotonia, developmental delay, and severe to profound cognitive impairment. The patient with the L909F mutation had initially refractory seizures and cerebellar ataxia, whereas the L208P patient had seizures only transiently but was overall more severely affected. In transfected mammalian cells, we determined the biophysical characteristics of L208P and L909F variants, relative to the wild-type channel and a previously reported gain-of-function Ca_v_3.1 variant. The L208P mutation shifted the activation and inactivation curves to the hyperpolarized direction, slowed the kinetics of inactivation and deactivation, and reduced the availability of Ca^2+^ current during repetitive stimuli. The L909F mutation impacted channel function less severely, resulting in a hyperpolarizing shift of the activation curve and slower deactivation. These data suggest that L909F results in gain-of-function, whereas L208P exhibits mixed gain-of-function and loss-of-function effects due to opposing changes in the biophysical properties. Our study expands the clinical spectrum associated with *CACNA1G* mutations, corroborating further the causal association with distinct complex phenotypes.

## 1. Introduction

Inherited variants in the *CACNA1G* gene were first suggested as risk alleles in families with idiopathic generalized epilepsy, although this still awaits definitive confirmation [1]. Pathogenic variants in *CACNA1G* were subsequently reported in familial spinocerebellar ataxia [2,3,4], a clinically heterogeneous neurodegenerative disorder [5]. In patients harboring the recurrent p.R1715H mutation and exhibiting spinocerebellar ataxia, the disease symptoms were primarily attributed to *CACNA1G* loss of function (LoF) and reduced neuronal excitability [2,3]. More recently, de novo mutations in *CACNA1G* were identified in some patients with childhood-onset cerebellar atrophy; clinical features were cerebellar ataxia and impaired cognitive development with other variable features, including epilepsy [6]. Among the patients with cerebellar atrophy, three displayed the recurrent p.A961T variant, whereas the fourth had the p.M1531V residue change [6]. Functional analyses of the variants suggested *CACNA1G* gain of function (GoF) as the disease mechanism [6]. However, it is plausible that the biophysical correlates of pathogenic *CACNA1G* variants associated with new aspects of severe developmental disorders do not fall simply into LoF or GoF categories.

*CACNA1G* encodes the pore-forming subunit of the low-voltage-activated (T-type) Ca_v_3.1 channel. This membrane protein is expressed in various areas of the CNS, showing particularly high levels in Purkinje neurons and the deep nuclei of the cerebellum [7]. Ca_v_3.1 channels typically exhibit low activation voltage, relatively small single channel conductance, and rapid inactivation upon opening [8,9]. They uniquely provide a significant source of calcium influx at subthreshold and suprathreshold voltage ranges [9,10,11]. At the synapses, Ca_v_3.1 channels directly or indirectly interact with other ion channels and modulate synaptic plasticity [12]. The activation of Ca_v_3.1 channels elicits low-threshold spikes, which in turn activate sodium channels and trigger the upstroke of action potentials. The overlap of the Ca_v_3.1channel’s activation and inactivation curves near the resting membrane potential results in a relatively large “window current”, allowing for regular/tonic firing, whereas channel activation from values more negative than the cell’s normal resting potential plays an important role in the genesis of rebound burst firing [10,11,13]. Targeting low-voltage-activated Cav3.1 channels is a suggested strategy for mitigating seizure activity [14]. 

In this study we characterized two missense *CACNA1G* variants, p. L208P and p. L909F, resulting in patients with an infantile-onset developmental and epileptic encephalopathy (DEE) without evident cerebellar atrophy. The biophysical properties of L208P and L909F Ca_v_3.1 channels were studied in human embryonic kidney (HEK) 293T cells. The M1508V Ca_v_3.1 channel variant, corresponding to the previously studied M1531V mutation underlying cerebellar atrophy [6], was also included as a positive control. Our results add to the current picture of *CACNA1G-*associated DEE by revealing new facets of clinical heterogeneity.

## 2. Results

### 2.1. Patients with CACNA1G Mutations

**Case 1:** This patient was previously reported in brief [15]. Pregnancy and birth were unremarkable and early milestones were normal (smiling; trying to sit). She had seizure onset at 7 months associated with hypotonia and developmental regression, evolving to severe global developmental delay. She exhibited seizures in the setting of fever, vibratory tonic seizures, tonic-clonic seizures, and absences. Seizures were refractory and bouts of severe seizures were associated with further regression. Truncal ataxia was evident on examination; she ambulated with a walker at age 4 years. Electroencephalography showed generalized spike-wave discharges, polyspike waves, multifocal discharges, and generalized paroxysmal fast activity (Figure S1). Brain MRI at 8 years was normal. Unlike previously reported cases [6], she had a milder phenotype. Dysmorphic features and cerebellar atrophy were absent. At age 9 years, she had been seizure free for 2 years on topiramate monotherapy. She showed eye gaze-based communication, co-operation with dressing, and independent finger-feeding. Whole-exome sequencing identified a c.2727G > C; p.L909F mutation in *CACNA1G*; due to a lack of paternal DNA, de novo status is unproven.

**Case 2:** This male was evaluated at age 17 years. Reduced fetal movements were noted at 34 weeks gestation, and he was delivered at 42 weeks via spontaneous vaginal delivery. No complications were noted in the neonatal period. Concerns regarding his development emerged at 2 months when he was noted to have significant head lag. He presented with myoclonic jerks at 4 months; at this time, he was noted to have generalized hypotonia, poor head control, and feeding difficulties. Antiepileptic medication was initiated at 4 months though EEG was normal at that time and events not captured. Subsequent EEGs were abnormal by report. EEG at age 1 year showed multifocal spikes and sharp waves and generalized bursts of 4-4.5 Hz activity and sharply contoured 6-7 Hz activity. Some of the discharges were associated with myoclonic jerks of the limbs. Anti-seizure medication (ASM) was discontinued at 4 years of age after a few years of no observable spells. He has not had any events concerning seizures since cessation of ASMs. Currently, at 17 years of age, the patient has profound cognitive impairment; he is unable to sit unsupported, is wheelchair-dependent, and has no speech, though his receptive language is somewhat spared and he communicates with eye gaze and making sounds. His neurological examination revealed significant axial hypotonia with appendicular spasticity and contractures of the hips, knees, ankles, and fingers. He never developed functional hand use and has intermittent bruxism. He is fed exclusively via a G-tube. In his adolescent years, he developed severe neuromuscular scoliosis and underwent surgical repair. Multiple brain MRIs have been normal. His prior genetic and metabolic workup was negative, including karyotype, array-based comparative genomic hybridization, Angelman syndrome testing, congenital disorders of glycosylation testing, *HEXA* gene analysis, *PLP1* gene sequencing, *SLC9A6* gene sequencing, peroxisomal studies, pipecolic acid, purines, pyrimidines, CSF amino acids, and basic metabolic studies. Muscle biopsy histology was normal. The patient underwent trio-based whole-exome sequencing on a clinical basis, which identified a de novo *CACNA1G* variant (NM_018896.4; c.623T>C; p.L208P).

### 2.2. Biophysical Characterization of Ca_v_3.1 Channel Variants

**Figure 1:**
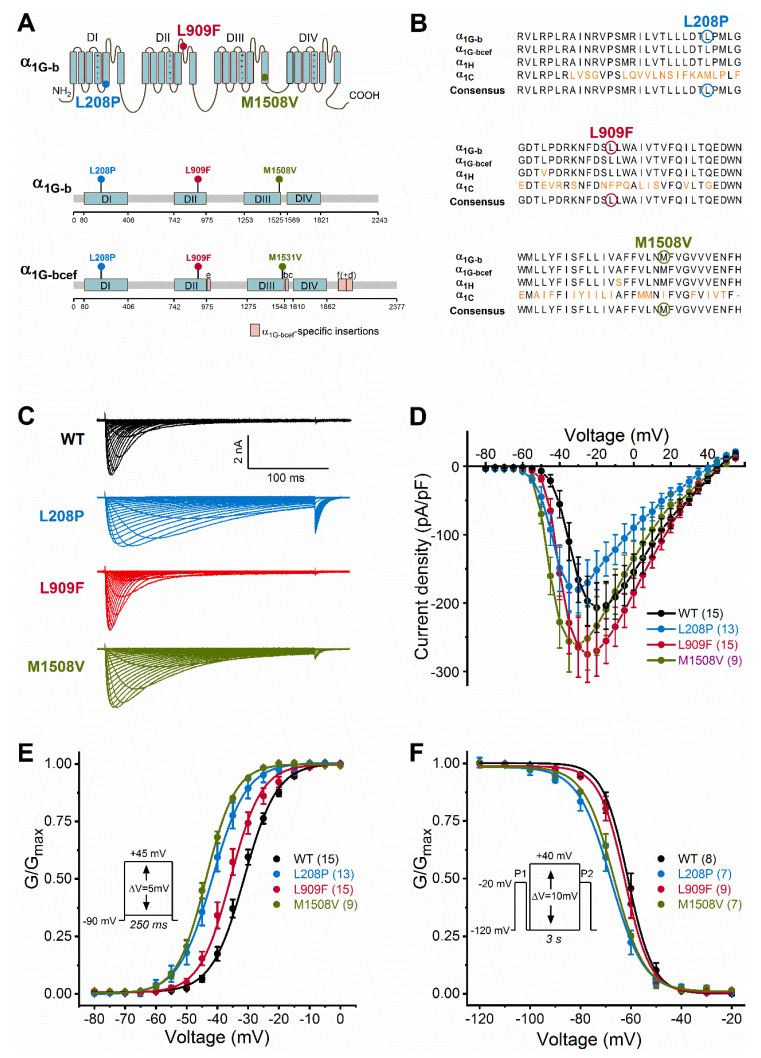
Ca_v_3.1 channel transmembrane topology, calcium channel homology, and voltage dependence of activation and inactivation of wild-type and mutant Ca_v_3.1 channels. (**A**), Location of Ca_v_3.1 channel mutations. The four membrane domains are labelled DI−DIV. The positive charges of segment 4 are shown in each domain. Note that all mutations fall in the pore region (segments 5 or 6). Lollipops representations of isoform b (α_1G-b_) and isoform bcef (α_1G-bcef_), showing the position of mutations. The M1508V mutation in α_1G-b_ corresponds to the M1531V mutation in α_1G-bcef_ [6]. Note the α_1G-bcef_-specific insertions in loop II-III (insertion e), loop III-IV (bc), and the C terminus (f and d) compared to α_1G-b_. (**B**), The L208P, L909F, and M1508V mutations affect highly conserved amino acid residues within the Ca_v_3 channel family. Sequence alignments of the α_1G-b_, α_1G-bcef_, α_1H_ (human Ca_v_3.2 channel), and α_1C_ (rabbit Ca_v_1.1 channel) regions affected by the mutations. Ca_v_1.1 was included in the analysis because of the known high-resolution structure of this channel [16]. The conserved sequence motifs nearby the mutations are shown; non-conserved residues are in orange; residues affected by mutations are marked by circles. (**C**), Representative whole-cell current traces are shown from top to bottom for the wild-type (WT), L208P, L909F, and M1508V variants. Current traces were elicited using the voltage protocol described in the material and methods and shown in E. (**D**), Current density–voltage relationships. Maximum inward current densities of individual Ca_v_3.1 variants were not significantly changed compared to WT (one-way ANOVA followed by Dunnett’s post-hoc test; Table 1). (**E**), Voltage dependence of activation. Normalized conductance–voltage relationships of G/G_max_ were obtained by nonlinear least-squares fits of data with Boltzmann equations (Equation (1)). The mean half-maximal activation voltages (V_0.5,act_) of L208P, L909F, and M1508V were significantly hyperpolarized compared to WT (in all cases *p* < 0.0001), whereas the slope factor (k) values were unchanged (one-way ANOVA followed by Dunnett’s post-hoc test; Table 1). (**F**), Voltage dependence of inactivation was assessed using the voltage protocol described in the material and methods and shown in the inset. Parameters of inactivation were obtained by fitting data with Boltzmann equations (Table 1). Relative to WT, the mean half-maximal inactivation voltages (V_0.5,inact_) of L208P and M1508V were significantly hyperpolarized (in both cases *p* < 0.0001), whereas that of L909F was unchanged; the k values of inactivation of all variants were not significantly different (one-way ANOVA followed by Dunnett’s post-hoc test; Table 1). For all variants, the shifts of the activation and inactivation curves resulted in an increased window current relative to WT, consistent with GoF. Data are mean ± SEM; n, the number of experiments in parentheses.

Similar to the missense M1531V and A961T Ca_v_3.1 variants associated with GoF and childhood-onset cerebellar atrophy [6], the L208P and L909F mutations affect the pore domain of the channel (Figure 1A). L208P, L909F, and M1508V mutations affect amino acids that are highly conserved in Ca_v_3.1 and Ca_v_3.2 channels. The alignment of human Ca_v_3.1 and rabbit Ca_v_1.1 channel protein sequences (Figure 1B) and the assessment of Ca_v_1.1 channel structure [16] reveal that the neutral leucine residue in position 208 (L208) is located at the distal end of the linker between segments S4 and S5, in domain I; whereas L909 is located in the ‘exon 12a’ region of the extracellular loop between S5 and S6, in domain II. In invertebrates, alternative splicing of exon12a affects the ionic selectivity [17].

**Figure 2:**
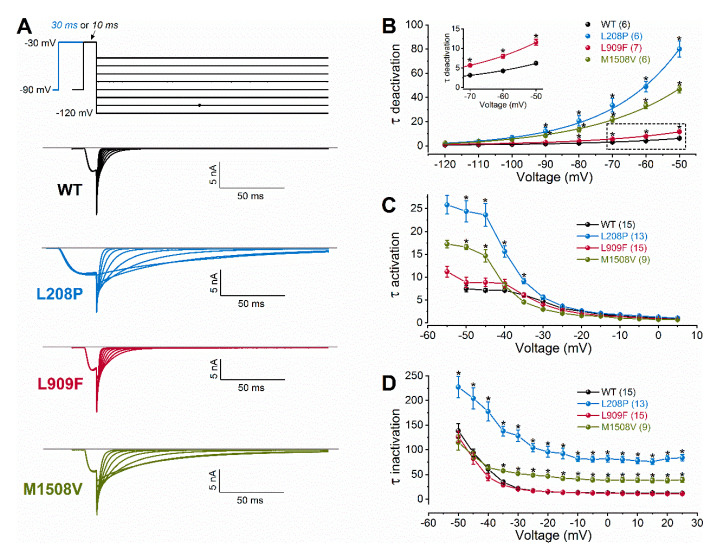
Voltage dependence of wild-type (WT) and mutant Ca_v_3.1 current kinetics. (**A**), Representative tail currents, elicited by 10- or 30-ms conditioning test pulses to −30 mV. Deactivation was recorded during subsequent repolarizations to test potentials from −120 to +50 mV, in 10-mV increments. For clarity, only current traces elicited by test potentials from −120 to −50 mV are shown (top: voltage protocol). Horizontal grey lines indicate zero-current levels. (**B**), Mean time constants of deactivation, resulting from fitting the deactivating current traces with a single-exponential function (Equation (2)). Inset shows boxed τ_deactivation_ values on an expanded scale. (**C**), Mean time constants of activation, obtained from fitting the inward current component underlying activation. (**D**), Mean time constants of inactivation, obtained from fitting the inactivating phase of the current. Data are mean ± SEM; the number of experiments is shown in parentheses; The *p* values of ‘Ca_v_3.1 variant ∗ voltage’ interaction associated with the time constants of activation, inactivation, or deactivation kinetics were < 0.0001. Asterisks indicate statistically significant differences compared to WT (*p* < 0.05 in two-way ANOVA followed by Dunnett’s post hoc test); see also Table 1.

**Figure 3:**
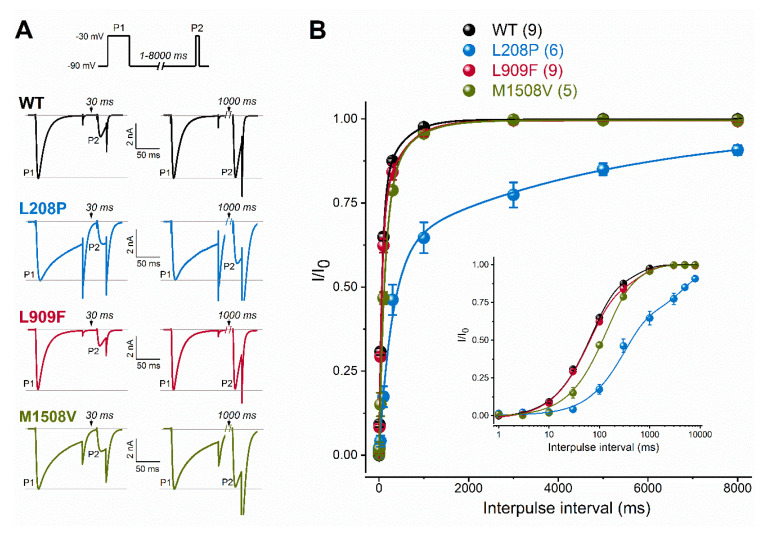
Recovery from fast inactivation of WT and mutant Ca_v_3.1 channels. (**A**), Recovery was evaluated with a paired-pulse protocol of depolarizing the cells for 100 ms (P1) and varying the interpulse interval (between 1 and 8000 ms) before the P2 pulse. Representative P1- and P2-elicited traces separated by 30 and 1000 ms recovery interpulse intervals (left). Horizontal grey lines indicate zero-current and P1-elicited maximum-current levels. (**B**), Plots of normalized WT and mutant peak calcium currents as a function of interpulse duration. (inset: same dataset with a logarithmic time scale). Data are mean ± SEM; the number of experiments, n, is shown between parentheses. The recovery time course could be described by two kinetic components (Equation (3)). Relative to WT, parameters of recovery remained largely unchanged except for the mean fast and slow time constants of the L208P variant (*p* < 0.0001 and *p* < 0.001, respectively, in one-way ANOVA followed by Dunnett’s post-hoc test; see Table 1).

We studied the biophysical properties of L208P, L909F, M1508V, and wild-type Ca_v_3.1 channel variants transiently expressed in HEK-293T cells. Whole-cell Ca^2+^ currents through L208P or M1508V channels displayed a slow inactivation time course compared to the wild type (Figure 1C; Table 1). The peak current densities of all variants were similar (Figure 1D; Table 1). In cells expressing L208P channels, the V_0.5,act_ and V_0.5,inact_ values displayed 10.4 and 6.7 mV hyperpolarizing shifts, respectively (Figure 1E,F; Table 1), slow channel kinetics over a range of depolarized membrane potentials (Figure 2), and markedly slow recovery from inactivation (Figure 3) relative to the wild type.

A hyperpolarizing shift of the activation curve and slow inactivation and deactivation kinetics can contribute to an increased inward Ca^2+^ current, consistent with GoF. Conversely, a slower activation time course of the current, a hyperpolarizing shift of the inactivation curve, and/or a slow recovery from inactivation can be associated with reduced current availability, consistent with LoF. Relative to the wild type, the L909F mutation mainly affected the V_0.5,act_, leading to a 4.5-mV hyperpolarizing shift, whereas the voltage dependence of inactivation remained unchanged (Figure 1E,F; Table 1). The deactivation of L909F channels was slow compared to the wild type, while the (in)activation kinetics and the time course of recovery from inactivation remained unaffected (Figure 2 and Figure 3; Table 1). Thus, in cells expressing L909F, the functional effects of the mutation are consistent with GoF. 

The biophysical properties of the M1508V variant were also determined. This variant was used as a comparator and corresponds to the previously characterized M1531V [6] (see the materials and methods and Figure 1A). Relative to the wild type, M1508V resulted in hyperpolarizing shifts of the activation and inactivation curves (Figure 1) and slower channel kinetics (Figure 2). The overall changes in the biophysical properties of M1508V were similar to those exhibited by M1531V [6], suggesting that the sequence differences between isoforms ‘b’ and ‘bcef’ (Figure 1A) did not modify the impact of this mutation. Any differences between the V_0.5,act_ or V_0.5,inact_ values of M1508V (Table 1) and those of M1531V [6] are likely due to the inherent differences between the voltage-dependent properties of isoforms b and bcef, and the liquid junction potentials, which were not corrected in our experiments but were adjusted in the study performed by Chemin et al. [6].

**Figure 4:**
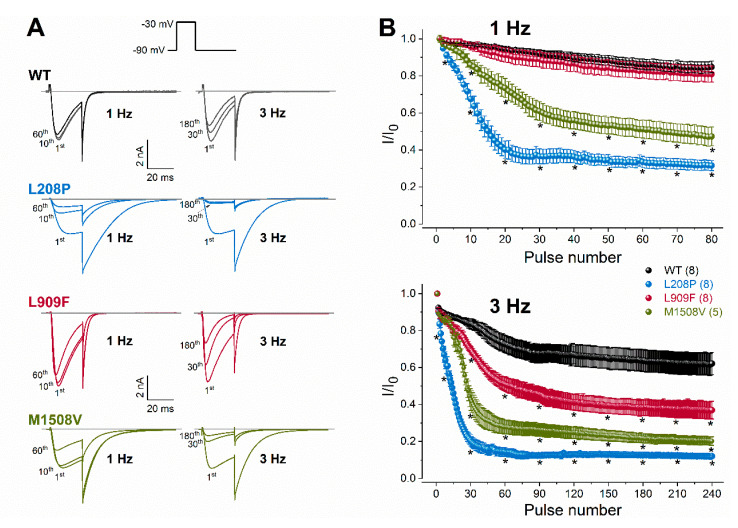
Rate-dependent changes in calcium current availability of WT and mutant Ca_v_3.1 channels. (**A**), Representative whole-cell currents recorded during the 1st, 10th, and 60th depolarization at 1 Hz, or during the 1st, 30th, and 180th depolarization at 3 Hz. Inset: voltage protocol. Horizontal grey lines indicate zero-current levels. (**B**), Plots of normalized WT and mutant peak calcium currents as a function of the stimulus number for the 1- (top) and 3-Hz (bottom) stimulus rates. Currents were normalized to the first stimulus. Data are mean ± SEM; the number of experiments, n, is shown in parentheses; Selected mean current amplitude values, elicited by pulse number 1, 3, 10, 20, 30, 40, 50, 60, 70, and 80 during 1-Hz stimulation and pulse number 1, 3, 10, 30, 60, 90, 120, 150, 210, and 240 during 3-Hz stimulation were included in the statistical analysis. The *p* values of the ‘Ca_v_3.1 variant ∗ pulse number’ interaction associated with current availability at 1 or 3 Hz were < 0.0001. Asterisks indicate statistically significant differences compared to WT (*p* < 0.05 in two-way ANOVA followed by Dunnett’s post hoc test); see also Table 1.

To evaluate channel availability during repeated depolarizations, wild-type or mutant channel currents were elicited by 25-ms test pulses at 1 or 3 Hz for 80 s (Figure 4). These frequencies were selected to mimic repetitive and/or burst action potential firing. Relative to the wild type, the availability of L909F currents during successive stimuli was similar at 1 Hz and reduced at 3 Hz. The availability of both L208P and M1508V currents at the end of the 1- and 3-Hz stimulus protocols was significantly reduced compared to the wild type (Figure 4; Table 1). Remarkably, the availability decrease for L208P was already apparent after 10 stimuli at 1 Hz. It is likely that the delayed recovery from inactivation exhibited by L208P (Figure 3) simultaneously contributes to the reduced availability.

Taken together, our data suggest that the L909F variant results in GoF due to the hyperpolarized shift of the activation curve and the delayed deactivation kinetics, whereas the L208P variant produces mixed GoF/LoF effects due to the opposing shifts of the activation and inactivation curves (GoF/LoF), the altered channel kinetics leading to increased current availability (GoF), the increased window current (GoF), and the slow recovery from inactivation (LoF) relative to wild-type channels. Brief depolarizations at 1 and 3 Hz reduce the L208P calcium current availability relative to the wild type, whereas the L909F variant displays reduced availability only in response to 3 Hz.

## 3. Discussion

The *CACNA1G* gene has been associated with various forms of cerebellar ataxia and neurological comorbidities [2,3,4,6,18,19]. Our study further validates *CACNA1G* in the group of pathogenic neuronal ion channel genes associated with DEE [20,21,22,23]. In both cases, the clinical features were compatible with those described in patients with childhood-onset cerebellar atrophy with severe motor and cognitive impairment, although dysmorphic features were not prominent in our cases [6]. Importantly, neither of our patients exhibited cerebellar atrophy. Ataxia, typical in cerebellar atrophy patients [6,24], was clearly identifiable in the L909F case, whereas in case 2 with L208P, motor development was never adequate to show definitive cerebellar signs. 

Our electrophysiological data are congruent with the clinical findings of a milder phenotype for the L909F patient versus the L208P (Table 1). The L909F mutation results in changes of the biophysical properties that are consistent with a gain of Ca_v_3.1 channel function. However, the L208P variant was associated with multiple and mixed biophysical defects, including gain of function due to a hyperpolarizing shift of the activation and a slowing of the inactivation and deactivation kinetics; and loss of function due to a hyperpolarizing shift of the inactivation, slow recovery from inactivation, and reduced availability due to enhanced entry to inactivation during repetitive stimuli.

In previous studies, computer modelling in deep cerebellar nuclei (DCN) neurons incorporating loss-of-function [3] or gain-of-function [6] Ca_v_3.1 channel variants resulted in decreased or increased neuronal excitability, respectively. Our results demonstrate that the biophysical properties of the L208P variant are similar to those of M1531V [6] causing enhanced firing activity in a DCN model cell [6]. Therefore, it is plausible that the L208P variant would also promote increased neuronal firing activity when implemented in the DCN model cell used previously [6,25]. It is expected that the L909F variant would also promote increased firing activity in this DCN neuron model. However, the full-scale functional impacts of Ca_v_3.1 channel variants may well depend on the physiological realism of the computational neuron. Hence, the neurophysiological consequences of the L208P mutation would likely be more complex in an improved virtual neuron, which, in addition to the parameter settings of the DCN neuron model, also incorporates enhanced entry into inactivation during repetitive stimulation and delayed recovery from inactivation processes. Fast recovery from inactivation is a critical biophysical characteristic enabling Ca_v_3.1 channels to participate in rebound burst depolarizations [10]. Therefore, it is plausible that L208P cannot support the burst-firing mode of neurons due to its reduced availability at a high stimulation rate relative to wild-type channels. Clearly, more work remains to be done in order to fully understand the neuron-scale mechanisms caused by pathogenic Ca_v_3.1 variants. In future studies, the use of a real-time dynamic clamp approach [26] should be considered to reveal the unique impacts of Ca_v_3.1 mutations on action potential firing and neuronal excitability.

Ca_v_3.1 channels are present at birth and show brain region- and age-dependent expression patterns; the overall expression levels typically increase throughout postnatal development [27,28]. Human brain transcriptomics data (https://www.proteinatlas.org) suggest that Ca_v_3.1 is expressed at high levels in the cerebellum, cerebral cortex, hypothalamus, and olfactory region. *CACNA1G* undergoes alternative splicing, generating unique Ca_v_3.1 channel isoforms exhibiting differential biophysical characteristics capable of fine-tuning neuronal excitability in the CNS [19,29,30,31]. Isoform b is normally found in the fetal brain, whereas the transcripts of the longer splice variants predominate in the adult brain [30]. In this study, the biophysical parameters of the wild-type Ca_v_3.1 isoform b were similar to the previously reported values [29] but slightly differed from those of isoform bcef [6]. However, the extent by which the M1508V mutation (isoform b) impacted Ca_v_3.1 function was highly similar to the effect of M1531V (isoform bcef).

Ca_v_3.1 channel density typically exhibits a somato-dendritic gradient, which could contribute to synaptic plasticity [32]. How neuronal plasticity is affected by *CACNA1G* mutations is currently unknown. *Cacna1g*-knockout mice seem to be less prone to absence epilepsy likely due to the absence of burst mode firing of thalamocortical relay neurons in response to membrane hyperpolarization [33,34]. Knockout of *Cacna1g* per se causes no detectable motor defects in mice. However, when a double knockout is created with a GABA_A_ receptor α1 subunit-null, a genetic model of essential tremor, severe motor coordination defects and loss of cerebellar Purkinje cells are observed [35]. It has been demonstrated that transgenic alteration of Cacna1g expression acts as a modifier of epilepsy in the Scn2aQ54 mouse model of focal epilepsy, with elevated levels of Cacna1g increasing spontaneous seizure frequency, whereas reduced Cacna1g diminishing seizures [36]. Similarly, in a mouse model of Dravet syndrome due to *Scn1a* mutation, decreased Cacna1g expression results in a partial amelioration of disease phenotypes with improved survival and reduced spontaneous seizure frequency [37]. These findings suggest that Cav3.1 may also be a potential molecular target for therapeutic intervention in *SCN1A* and *SCN2A* epilepsy patients. In future studies, transgenic mice and/or stem cell modeling could also serve as useful tools for the investigation of *CACNA1G* variants.

Ca_v_3.1 channels are expressed in the embryonic hearts of various mammals, but expression decreases during development [10,38]. In rodents, the functional role of Ca_v_3.1 has been demonstrated. Mice lacking Ca_v_3.1 channels exhibit decreased pacemaker activity, corroborating further atrioventricular conduction compared with the wild type [39]. Ca_v_3.1 mRNA and protein has also been detected in the conduction system of the adult human heart [40]; however, a Ca_v_3.1-specific Ca^2+^ current has not yet been identified in human atrial, ventricular, or sinoatrial node cells [41]. Therefore, it is not surprising that the cardiac function and ECG data of patients carrying the L208P and L909F mutations were normal. 

In conclusion, our study expands the clinical spectrum associated with *CACNA1G* mutations, and the causal link between Ca_v_3.1 channel dysfunction and distinct complex phenotypes. In two patients, Ca_v_3.1 channel dysfunction correlates with phenotype severity in infantile-onset developmental and epileptic encephalopathy without cerebellar atrophy.

## 4. Materials and Methods 

### 4.1. Patients 

The study was approved by the Human Research and Ethics Committee (Tasmania) Network (Reference H0013627; approval 25 November 2013) and by the Institutional Review Board of the Children’s Hospital of Philadelphia (Reference 15-012226, approval 6 November 2015). The L909F mutation was reported as a likely pathogenic variant in our recently published study [15]. The L208P mutation was identified using the GeneMatcher website at http://www.genematcher.org [42]. Case 2 (L208P) underwent trio-based whole-exome sequencing through GeneDx, as previously described [43]. Written informed consent for patient data inclusion was obtained from the parents or legal representatives of the patients in the study. Anonymized data will be shared by request from any qualified investigator.

### 4.2. Ca_v_3.1 Channel Clones, Mutagenesis, and Tools for Bioinformatics

The human wild-type *CACNA1G* cDNA construct, encoding low-voltage-activated human Ca_v_3.1 channel isoform b (NCBI accession AF126965.1), was provided by Dr. Gerald W. Zamponi (Department of Physiology and Pharmacology, University of Calgary, Canada). Isoform b is shorter than isoform bcef (also known as variant 1; NM_018896.5), which was used previously to assess the impact M1531V mutation on Ca_v_3.1 function [6]. More specifically, isoform b lacks four insertions relative to isoform bcef, including ‘e’ (23 amino acids) within the II-III loop, ‘bc’ (18 amino acids) within the III-IV loop, ‘f’ (48 amino acids), and the adjacent ‘d’ (45 amino acids) in the C-terminal region (nomenclature according to Monteil et al. [44]). The c.623T>C p.L208P, c.2727G>C p.L909F, and c.4522A>G p.M1508V mutations were introduced into isoform b, using a QuikChange Lightning Site-Directed Mutagenesis Kit (Agilent Technologies, Santa Clara, CA). Thus, the p.M1508V mutation in isoform b corresponds to the c.4591A>G p.M1531V mutation in the longer isoform ‘bcef’ [6]. Custom oligonucleotides were obtained from Bioneer Pacific (Kew East, Victoria, Australia):

L208P-forward (f), CTGCTGGATACGCCGCCCATGCTGGGC;
L208P-revers (r), GCCCAGCATGGGCGGCGTATCCAGCAG;
L909F-f, GGAAGAATTTTGACTCCTTCCTCTGGGCCATCG;
L909F-r, CGATGGCCCAGAGGAAGGAGTCAAAATTCTTCC;
M1508V-f, CCTTCTTTGTCCTGAACGTGTTTGTGGGTGTGGTG;
and M1508V-r, CACCACACCCACAAACACGTTCAGGACAAAGAAGG.

All clones were verified by automated DNA sequencing (Australian Genome Research Facility, Melbourne, Victoria, Australia). Selected regions of isoform b were compared with the corresponding homologous regions of v1 [6], human Ca_v_3.2 channel (NCBI accession NP_066921.2), and rabbit Ca_v_1.1 channel (NCBI accession NM_001101720.1) using CLC Sequence Viewer 7.7 (QIAGEN, Denmark). Lollipop diagrams were created using Lollipops software available at https://github.com/pbnjay/lollipops.

### 4.3. Heterologous Expression of Ca_v_3.1 Channel Variants and Electrophysiology

Human embryonic kidney-293T cells expressing SV40 large T antigen (HEK293T) were cultured as previously described [45]. The cells were transiently co-transfected with plasmids encoding wild-type, L208P, L909F, or M1508V Ca_v_3.1 channel variant (2 μg) and enhanced green fluorescent protein (eGFP; 1 μg; Clontech, Mountain View, CA), using Lipofectamine 3000 Reagent (Thermo Fisher Scientific (Scoresby, Victoria, Australia). After transfections, cells were incubated at 37°C in 5% CO_2_; 48 h post transfection, the cells were detached using TrypLE Express Reagent (Thermo Fisher Scientific) and plated on Menzel-Gläser glass coverslips (Thermo Fisher Scientific). 

Electrophysiological recordings were performed within four days post transfection at room temperature (23 ± 0.5 °C). Fire-polished borosilicate patch pipettes (Harvard GC150TF-7.5 from SDR Scientific, Chatswood, New South Wales, Australia) were pulled using a Sutter P-1000 micropipette puller (Sutter Instruments, Novato, CA) and typically exhibited resistance values of 1.5 MΩ. Whole-cell Ca^2+^ currents were recorded using a Multiclamp 700B amplifier (Molecular Devices, Sunnyvale, CA) controlled by a pCLAMP 9/DigiData 1440 acquisition system (Molecular Devices). Cells were superfused with an extracellular solution containing 110 mM NaCl, 3 mM CaCl_2_, 1 mM MgCl_2_, 5 mM CsCl, 30 mM TEA-Cl, 10 mM D-glucose, and 10 mM HEPES (pH adjusted to 7.4 with TEA-OH), at a constant rate of ~0.5 mL/min; the intracellular (pipette) solution contained 125 mM K-gluconate, 2 mM MgCl_2_, 5 mM EGTA, 5 mM NaCl, 2 mM Na_2_ATP, 2 mM Phosphocreatine-Na_2_, and 10 mM HEPES (pH adjusted to 7.25 with CsOH). Currents were low-pass filtered at 10 kHz and digitized at 50 kHz. Series resistance compensation was > 80% in all cases. The membrane voltage values were not corrected for the estimated liquid junction potential of ~14 mV. Leak and capacitive currents were corrected using a −P/4 pulse except when determining steady-state inactivation, recovery from inactivation, and rate-dependent changes of the peak current.

### 4.4. Curve Fitting and Statistical Analysis 

Data were analyzed off-line using Clampfit 9.2 (Molecular Devices) and OriginPro (Microcal Software Inc., Northampton, MA). Peak current, current density, voltage dependence of activation and inactivation, recovery from inactivation, and the stimulation rate dependence of the peak current were determined as previously described [46]. The voltage protocols are described in the results and are also shown as inserts in the figures. For most protocols, a holding potential (HP) of −90 mV was used, whereas the voltage-dependence inactivation was determined from an HP value of −110 mV. Briefly, current–voltage (I−V) relationships were determined using peak Ca^2+^ currents elicited by depolarizing voltage steps in 5-mV increments between −80 and +50 mV, at 0.333 Hz. Ca^2+^ current was converted to peak conductance by the equation G = I/(V-V_rev_), where V_rev_ is the reversal potential. Normalized conductance–voltage relationships were plotted as G/G_max_ values versus voltage and are referred to as activation curves. The voltage dependence of inactivation was obtained using a 30-ms P1 pulse (control) to −20 mV, followed by 3-s pre-pulses in 10-mV increments in the voltage range between −120 and +10 mV, and a subsequent 30-ms P2 pulse (test) to −20 mV. Inactivation values were obtained by calculating the P2/P1 ratio. Activation and inactivation curves were fit using the Boltzmann equation:

(1) $$\frac{G}{G_{\max}}=\frac{1}{\left[1+e^{\left(V-V_{0.5}\right)/k}\right]}$$

where V is the conditioning voltage, V_0.5_ is the half-maximal (in)activation voltage, and k is a slope factor. The voltage dependence of the time constants of peak Ca^2+^ current activation, inactivation, and deactivation were obtained by fitting the time course of individual current traces with a single-exponential equation:

(2) $$\frac{I}{I_{\max}}={\mathrm{Ae}}^{-t/\tau }$$

where t is the time, A is the amplitude, and τ is the time constant. The τ of activation (τ_activation_) was plotted against the test potential in the range between −60 and +5 mV; the τ of inactivation (τ_inactivation_) was plotted against the test potential in the range between −50 and +25 mV, whereas the τ of deactivation (τ_deactivation_) was plotted against the test potential in the range between −120 and −50 mV. Voltage dependence of recovery from (fast) inactivation was assessed using a paired-pulse protocol comprising a 100-ms pre-pulse to −30 mV (P1), which served to fast-inactivate the I_Na_, followed by a test pulse to −30 mV (P2), to measure Ca^2+^ current availability after variable recovery intervals between 1 and 8000 ms. Recovery was analyzed by fitting a double-exponential function to the data to obtain the ‘fast’ and ‘slow’ time constant, τ_f_ and τ_s_, respectively:

(3) $$\frac{I}{I_{\max}}=A_{f}\left(1e^{-t/\tau_{f}}\right)+A_{s}\left(1e^{-t/\tau_{s}}\right)$$

where t is time (here the delay between pre-pulse P1 and test pulse P2), and A_f_ and A_s_ are the amplitudes of the fast and slow component of recovery, respectively. The rate of entry into slow inactivation that developed during 25-ms depolarizations at 1 or 3 Hz was estimated from the peak Ca^2+^ current decrease during 60 or 180 depolarizing pulses, at 1 and 3 Hz, respectively.

Data are presented as mean ± standard error of the mean (SEM); n, number of experiments. Statistical analyses, including one-way analysis of variance (one-way ANOVA) or two-way ANOVA, followed by pairwise comparison using the Dunnett’s multiple comparison test, were performed using GraphPad Prism version 8 (La Jolla, CA, USA). *p* values <0.05 were considered statistically significant.

## Figures and Tables

**Table 1:** Biophysical parameters of Ca_v_3.1 channel variants expressed in HEK-293T cells.

Variant \\ Biophysical Property	Wild-Type	L208P	L909F	M1508V
Current density (pA/pF) n	206.9 ± 36.2 (15) 15	180.5 ± 37.4 (13) 13	275.2 ± 40.8 (15) 15	261.5 ± 46.9 (9) 9
Activation V_0.5,act_ (mV) k_act_ (mV) n	−31.27 ± 0.44 5.33 ± 0.21 15	−41.66 ± 0.50 **** 5.56 ± 0.31 13	−35.72 ± 0.60 ****5.04 ± 0.2515	−43.68 ± 0.12 **** 4.81 ± 0.11 9
Inactivation V_0.5,inact_ (mV) k_inact_ (mV) n	−61.18 ± 1.01 5.84 ± 0.42 8	−67.93 ± 0.47 **** 6.50 ± 0.41 7	−62.43 ± 0.24 6.01 ± 0.31 9	−66.63 ± 0.33 **** 6.27 ± 0.29 7
Activation kinetics τ at −50 mV (ms) τ at −30 mV (ms) n	7.49 ± 0.73 4.72 ± 0.35 15	24.40 ± 2.35 **** 5.60 ± 0.44 13	8.84 ± 1.14 4.03 ± 0.28 15	16.58 ± 0.58 **** 2.99 ± 0.16 9
Inactivation kinetics τ at −50 mV (ms) τ at −10 mV (ms) n	138.49 ± 14.62 13.56 ± 0.58 15	227.32 ± 22.0 **** 81.90 ± 5.4 **** 13	126.20 ± 11.0 13.07 ± 0.59 15	114.91 ± 15.6 41.16 ± 3.8 ** 9
Deactivation kineticsτ at −70 mV (ms)τ at −50 mV (ms)n	3.11 ± 0.10 6.21 ± 0.27 6	33.55 ± 5.54 **** 80.04 ± 6.58 **** 6	5.66 ± 0.35 **** 11.6 ± 0.76 **** 7	21.91 ± 2.30 ** 46.72 ± 2.80 **** 6
Recovery^§^ τ_f_ (ms) A_f_ (ms) τ_s_ (ms) A_s_ (ms) n	66.52 ± 8.7 0.79 ± 0.07 450.03 ± 117.6 0.21 ± 0.07 9	299.82 ± 70.6 **** 0.61 ± 0.09 4904.4 ± 1608 *** 0.36 ± 0.06 6	61.05 ± 5.0 0.74 ± 0.04 492.28 ± 109.8 0.27 ± 0.04 9	112.18 ± 18.5 0.76 ± 0.12 534.03 ± 236 0.26 ± 0.13 5
Current availability 1 Hz, 10^th^ stimulus (%)− 1 Hz, 60^th^ stimulus (%)− 3 Hz, 30^th^ stimulus (%)− 3 Hz, 180^th^ stimulus (%)− n	0.969 ± 0.01 0.864 ± 0.03 0.835 ± 0.03 0.635 ± 0.06 8	0.597 ± 0.05 **** 0.335 ± 0.03 **** 0.205 ± 0.03 **** 0.125 ± 0.01 **** 8	0.958 ± 0.03 0.829 ± 0.04 0.695 ± 0.02 ** 0.382 ± 0.04 **** 8	0.859 ± 0.02 * 0.514 ± 0.05 **** 0.437 ± 0.06 **** 0.221 ± 0.02 **** 5

Data are represented as mean ± SEM; n, number of experiments, V_0.5,(in)act_, membrane potential for half-maximal (in)activation; k_(in)act_, slope factor of steady-state (in)activation curve; t_f_ and t_s_, fast and slow recovery time constants, respectively; A_f_ and A_s_, fractions of fast and slow recovery from inactivation, recovery**^§^**, recovery from fast inactivation. Two-way ANOVA with Dunnett’s multiple comparison test was used to statistically evaluate ‘current density’; time constants of activation, inactivation, and deactivation; and peak current amplitudes during 1- and 3-Hz depolarizations. One-way ANOVA with Dunnett’s multiple comparison test was used to evaluate the parameters of (in)activation (V_0.5,(in)act_, k_(in)act_) and recovery (t_f_, t_s_, A_f_, and A_s_). Note that the M1508V and L208P mutations cause similar changes of the V_0.5,(in)act_ values, time course of (in)activation, deactivation kinetics, and current availability relative to WT whereas the L909F mutation affects a smaller number of biophysical parameters relative to wild-type; Asterisks indicate statistically significant differences using one-way ANOVA or two-way ANOVA followed by Dunnett’s multiple comparison test; * *p* < 0.05, ** *p* < 0.01, *** *p* < 0.001, or **** *p* < 0.0001 compared with wild type.

## Supplementary Materials

Supplementary materials can be found at https://www.mdpi.com/1422-0067/21/17/6333/s1. Figure S1 shows clinical EEG recordings for case 1.
