# Supplementary material for: Novel Missense CACNA1G Mutations Associated with Infantile-Onset Developmental and Epileptic Encephalopathy
Source: Int J Mol Sci. 2020 Aug 31;21(17):6333. doi: 10.3390/ijms21176333 (PMC7503748; doi:10.3390/ijms21176333)
Supplement: Supplementary file 1 [file ijms-21-06333-s001.pdf]

Supplementary Materials

Novel missense *CACNA1G* mutations associated with infantile-onset developmental and epileptic encephalopathy

Géza Berecki, Katherine L. Helbig, Tyson L Ware, Bronwyn Grinton, Cara M. Skraban, Eric D. Marsh, Samuel F. Berkovic and Steven Petrou

Case 1 (*CACNA1G* variant c.2727G>C; p.L909F)

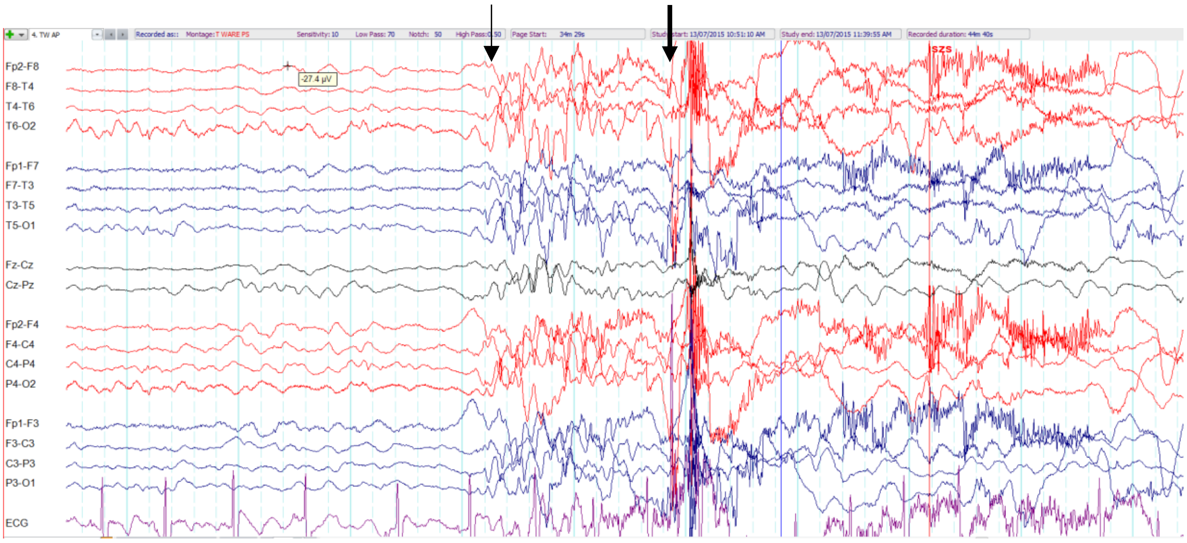

Figure S1. Electroencephalogram (EEG) of case 1 at age 5 years showing a burst of irregular generalized polyspike wave discharges (thin arrow) followed by a brief tonic seizure with generalized paroxysmal fast activity (thick arrow) contaminated by muscle artefact.
